# A microprocessor based on a two-dimensional semiconductor

**DOI:** 10.1038/ncomms14948

**Published:** 2017-04-11

**Authors:** Stefan Wachter, Dmitry K. Polyushkin, Ole Bethge, Thomas Mueller

**Affiliations:** 1 Institute of Photonics, Vienna University of Technology, Gußhausstraße 27-29, 1040 Vienna, Austria; 2 Institute of Solid State Electronics, Vienna University of Technology, Floragasse 7, 1040 Vienna, Austria

## Abstract

The advent of microcomputers in the 1970s has dramatically changed our society. Since then, microprocessors have been made almost exclusively from silicon, but the ever-increasing demand for higher integration density and speed, lower power consumption and better integrability with everyday goods has prompted the search for alternatives. Germanium and III–V compound semiconductors are being considered promising candidates for future high-performance processor generations and chips based on thin-film plastic technology or carbon nanotubes could allow for embedding electronic intelligence into arbitrary objects for the Internet-of-Things. Here, we present a 1-bit implementation of a microprocessor using a two-dimensional semiconductor—molybdenum disulfide. The device can execute user-defined programs stored in an external memory, perform logical operations and communicate with its periphery. Our 1-bit design is readily scalable to multi-bit data. The device consists of 115 transistors and constitutes the most complex circuitry so far made from a two-dimensional material.

## Abstract (web summary)

Two-dimensional materials are receiving increasing interest as they could pave the way to a paradigm shift in nano-electronics. Here, the authors demonstrate a 1-bit implementation of a microprocessor consisting of 115 transistors, using atomically thin MoS_2_.

Two-dimensional (2D) materials, such as semiconducting transition metal dichalcogenides (TMDs)^1^,^2^, black phosphorus^3^, silicene^4^ and others, are considered promising candidates for future generations of electronic circuits. It is currently not foreseen that silicon will be replaced for mainstream digital electronics in the mid-term future; however, similar to organic semiconductors^5^ or carbon nanotubes^6^, 2D materials offer a number of interesting properties that could lead to novel applications. Their ultrathin channel thickness provides improved electrostatic gate control and reduced short-channel effects^7^,^8^, which ultimately results in better geometric scaling behaviour^9^,^10^ and less power consumption. 2D semiconductors are also one of the leading candidates to enable tunnel field-effect transistors (FETs)^11^,^12^, working with sub-threshold swing below 60 mV per decade and thus low supply voltage. Together, with their high mechanical flexibility and stability, optical transparency, excellent optoelectronic properties^13^ and compatibility with standard semiconductor technology processing this could lead to energy efficient and flexible electronics^14^,^15^,^16^.

The field of TMD-based electronics has progressed enormously during the past few years. Soon after the first realizations of bulk^17^,^18^ and monolayer^2^ FETs, basic electronic circuits were demonstrated^19^,^20^. Both n-type (NMOS)^19^,^20^,^21^ and complementary (CMOS)^22^,^23^ metal-oxide–semiconductor technologies have been developed and a good understanding of the FET device physics has been gained^24^,^25^,^26^. The work on devices has been paralleled by the development of growth techniques^27^,^28^,^29^,^30^ for the large-scale fabrication of TMD films with good uniformity over the size of a wafer^30^ and the development of technologies for transferring 2D materials onto bendable^14^,^15^,^16^ substrates. Nevertheless, due to the plethora of challenges being faced in large-scale integration, previous work has so far been restricted to applications consisting of only a few transistors and with limited functionality. These challenges range from the necessity to match voltage levels and achieve high noise margins in cascaded logic stages to stringent requirements on device uniformity over millimetre size dimensions.

Here, we demonstrate the feasibility of using a 2D semiconductor to realize a complex digital circuit—a microprocessor.

## Results

### Microprocessor architecture

**Figure 1:**
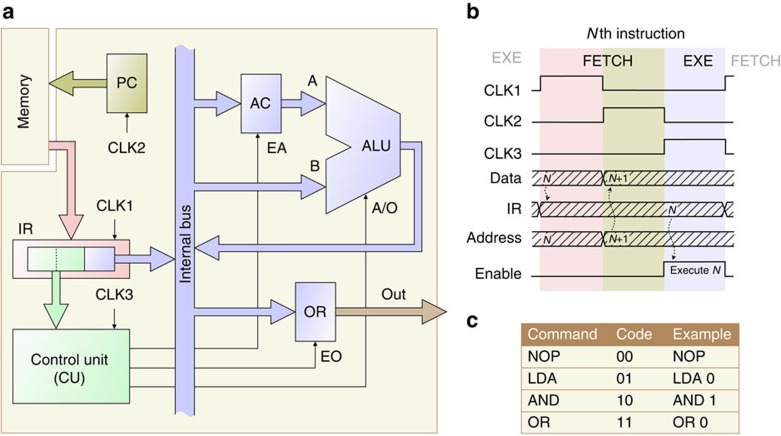
Microprocessor architecture. (**a**) Block diagram, showing the arithmetic logic unit (ALU) with inputs A and B, accumulator (AC), control unit (CU), instruction register (IR), output register (OR) and program counter (PC). Enable signals (EA and EO) and operation selection code (A/O) are supplied by the CU to the respective subunits. CLK signal generation and memory are implemented off-chip. (**b**) Timing diagram for the *N*th instruction cycle. During the FETCH sequence the content of the memory is loaded into the IR and the address, stored in the PC, is increased. During the EXE sequence the command, stored in the IR, is executed. (**c**) Instruction set of the microprocessor. NOP is the no-operation instruction; LDA transfers data from the memory into the AC; AND and OR perform logical operations.

Figure 1a depicts the architectural block diagram of our microprocessor. For demonstration purposes, we minimized transistor count and thus realized a device that operates on single-bit data only. We stress that this is not a fundamental limitation and the device is readily scalable to *N*-bit data, broadly speaking by connecting *N* of our devices in parallel. Although we reduced the architecture of our device to the essentials, it comprises all basic building blocks that are common to most microprocessors. In particular, these are: an arithmetic logic unit (ALU), that forms the heart of the processor and is, in general, capable of performing basic arithmetic and logical operations; for simplicity, we have implemented here only logical conjunction and disjunction operations. An accumulator (AC), which holds one of the operands to be supplied to the ALU. An instruction register (IR), that stores the content of the program memory currently being executed, where the most significant two bits contain the instruction itself and the third bit contains the data (Although we retrieve the data directly from the program memory, our device can also process data stored in a separate data memory (Harvard architecture). In this case, the IR is supplied with an address that points to the data memory content, which is then placed on the bus.). A control unit (CU), that receives as input the instruction code from the IR and orchestrates all resources by enabling components to access the internal bus via the control signals EA and EO; A/O conveys to the ALU the operation selection code (conjunction, A/O=0; disjunction, A/O=1). A program counter (PC), which supplies the memory with the address of the active instruction. And, finally, an output register (OR), that allows the processor to transfer the results of a calculation to the output port. The memory is, as usual, implemented off-chip.

Figure 1b depicts the timing diagram of the device, using three clock (CLK) signals. The execution of each instruction occurs in two sequences—a FETCH sequence followed by an execute (EXE) sequence. The FETCH sequence consists of two phases: in a first phase, the content of the external memory (at the address stored in the PC) is loaded into the IR; the PC is then incremented in a second step. During the EXE sequence, which is implemented here in a single phase, the microprocessor decodes and executes the command stored in the IR. This cycle is repeated continuously. Each phase is triggered by a CLK signal (CLK1, phase 1; CLK2, phase 2; CLK3, phase 3). In order to be flexible in terms of clock rate and timing, we generated the CLK signals externally; an on-chip implementation is straightforward. Figure 1c summarizes the instruction set that we have implemented. The instructions are encoded with two bits; some of them are followed by one bit of data. The no-operation (NOP) instruction has no effect other than to increase the PC. LDA allows the transfer of data from the memory into the AC. AND and OR perform logical conjunction and disjunction operations, respectively.

It is instructive to consider a simple example. The program fragment


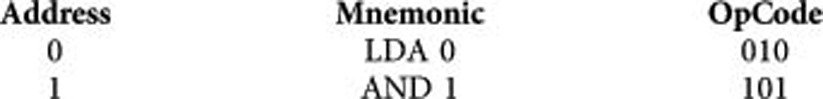


transfers in a first step, triggered by CLK1, the bit sequence 010 from the memory into the IR. CLK2 then increases the PC and the next instruction becomes available, but is not loaded into the IR yet. Triggered by CLK3, the CU then signals the AC (EA=1) to receive the data (0) from the IR via the internal bus. With the next CLK1 signal, the content of the IR is updated (IR=101), and the CU enables the ALU to perform a logical conjunction operation (A/O=0) between the data on the bus (1) and that stored in the AC during the previous instruction. Triggered by CLK3, the result of this operation (0) is finally written into the OR (EO*=*1).

### Device implementation

We now come to the actual device implementation using a 2D semiconductor. Our microprocessor was fabricated in gate-first technology on a silicon wafer with 280-nm-thick silicon dioxide. The substrate fulfills no other function than acting as a carrier medium and could thus be replaced by glass^31^ or any other material, including flexible substrates^14^,^15^,^16^. We fabricated 18 devices per wafer, with FET channels made from chemical vapour deposition (CVD) grown large-area bilayer MoS_2_ films. Two Ti/Au metal layers were used to interconnect the transistors and Al_2_O_3_ was used as gate oxide. A detailed description of the device fabrication steps can be found in Methods. Subunits, such as for example, the ALU or the IR, were provided with metal pads for individual testing in a wafer probe station. All subunits were eventually bonded together and the sample was placed back into the probe chamber, where it remained in vacuum for final testing of the complete circuit.

**Figure 2:**
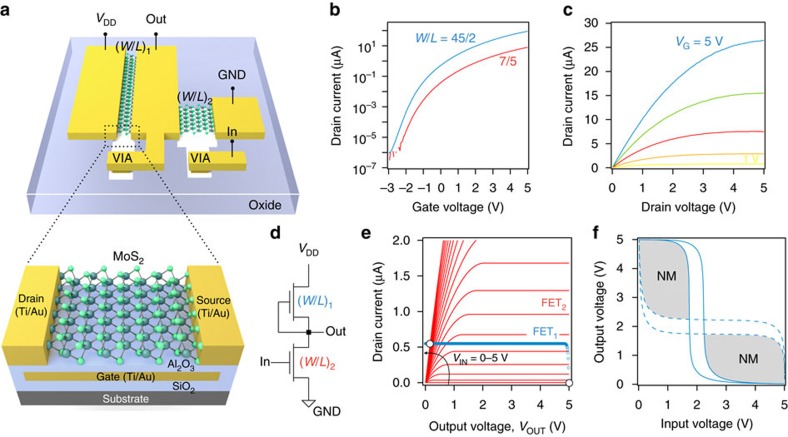
Characterization of MoS_2_ transistors and inverter. (**a**) Schematic drawing of an inverter circuit (top) and an individual MoS_2_ transistor (bottom) in gate-first technology (see Supplementary Fig. 5 for corresponding micrograph). (**b**) Transfer characteristics of load (*W*/*L*=45/2) and pull-down (*W*/*L*=7/5) transistors. (**c**) Output characteristic for gate voltages between 1 and 5 V (in 1 V steps). (**d**) NMOS inverter circuit schematic. (**e**) Graphical construction to determine the output voltage *V*_OUT_ of an inverter for a given input voltage *V*_IN_. The blue symbols show the load curve and the red lines are the output characteristics of the pull-down transistor (in 0.25 V steps). The intersection point of both curves determines *V*_OUT_. (**f**) The solid line shows the measured voltage transfer characteristic of an inverter. By mirroring this curve (dashed line) a butterfly plot is obtained, from which NM can be extracted by nesting the largest possible square in the grey shaded area.

Figure 2a (bottom) shows a schematic drawing of a so-obtained MoS_2_ FET. The devices exhibit a field-effect mobility of ∼3 cm^2^ V^−1^ s^−1^, a threshold voltage *V*_T_ of ∼0.65 V (Supplementary Fig. 3), an on/off ratio of ∼10^8^, and uniform behaviour over a ∼50 mm^2^ area over the wafer (Supplementary Fig. 4). The circuit is based on the NMOS logic family, where both pull-up (load) and pull-down networks were realized using n-type enhancement-mode FETs. The implementation of an inverter (see circuit schematic in Fig. 2d) using this logic family is shown in Fig. 2a (top). A careful design of the *W*/*L* ratios, where *W* and *L* denote the width and length of the FET channels, is crucial, as it determines the switching threshold voltage *V*_M_ and thus the ability to cascade logic stages. For simple analytic modelling, we performed calculations based on long-channel FET theory^32^. The pull-down FET is described by 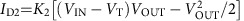 in the triode regime and 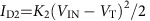 in the saturation regime (red curves in Fig. 2e). The load FET is operated in the sub-threshold regime (*V*_G1_=0<*V*_T_), and thus acts as a current source over a large drain voltage range, 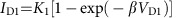 with *β* being the reciprocal of the thermal potential. From the circuit schematic Fig. 2d, it is apparent that 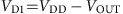, and thus 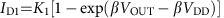 (blue symbols in Fig. 2e). The parameters *K*_1_ and *K*_2_ are taken from the experiment (Fig. 2b). By equating both currents, 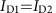, we obtain a relation between *V*_OUT_ and *V*_IN_, from which the switching threshold *V*_M_ can be determined (Supplementary Fig. 6). If both transistors are implemented with same *W*/*L* ratio, *V*_M_ drops below 1 V (Supplementary Fig. 6b), resulting in low noise margin, especially in the presence of additional hysteresis. Asymmetric transistor design, on the other hand, allows shifting *V*_M_ towards *V*_DD_/2 (Supplementary Fig. 6a), resulting in improved switching behaviour. *W*/*L* ratios of the pull-up and pull-down transistors were hence made 45/2 (μm/μm) and 7/5, respectively.

Logic NAND gates with *M* inputs were implemented by connecting *M* pull-down transistors with *W*/*L*=(*M* × 7)/5 in series. The processor was realized by using a combination of these elements. The minimum feature size of 2 μm was chosen rather large for two reasons. It makes the design immune to sample inhomogeneities (for example, small holes, cracks and contaminations in the MoS_2_ film) and also allows for fast visual inspection of the lithographic structures with an optical microscope. Because of the immunity of 2D transistors to short-channel effects^7^,^8^,^9^,^10^, we expect comparable performance when the devices are scaled to sub-micrometre dimensions, provided that low contact resistance can be achieved.

Figure 2b shows the transfer characteristics of load and pull-down transistors, where the ∼14 times higher current through the former demonstrates reliable controllability of the device characteristics by geometrical scaling. The output characteristic, depicted in Fig. 2c, shows clear current saturation due to channel pinch-off at the drain. The voltage transfer characteristic of our inverters exhibit excellent performance for a wide supply voltage range between *V*_DD_=2 and 7 V, with input and output logic levels being perfectly matched. Figure 2f (solid line) shows the results for *V*_DD_=5 V, for which the voltage gain 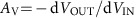 reaches values of *A*_V_≈60. Although the voltage transfer curve shows some hysteresis (that mostly stems from trap charges in the gate oxide) the noise margin of the inverter (see shaded area in Fig. 2f), NM≈0.59 × (*V*_DD_/2), is sufficiently large for integration into multi-stage logic circuits. The NAND gates showed comparable performance. We estimate a static power consumption of 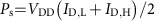
≈1.4 μW per logic gate, where *I*_D,L_ and *I*_D,H_ denote the currents at *V*_IN_=0 and 5 V (Fig. 2e), respectively. The total power consumption of the circuit, consisting of 41 stages, is thus ∼60 μW.

**Figure 3:**
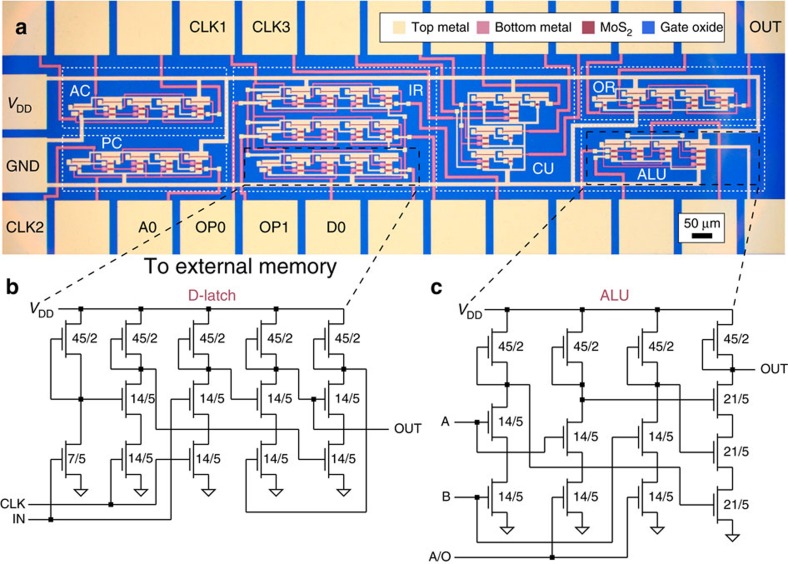
Device implementation using a 2D semiconductor. (**a**) Microscope image of the microprocessor. The two metal layers appear in different colour and are connected with via-holes. All subunits were provided with metal pads for individual testing. Labelled pads were used to connect the device to the periphery (memory, CLK signal generation, power supply, output), the others were wire bonded together to realize the internal connections. Scale bar, 50 μm. Circuit schematics of (**b**) D-Latch and (**c**) ALU, with *W*/*L* ratio in units of μm/μm for each transistor. IN, input; OUT, output. The complete microprocessor schematic is presented in Supplementary Fig. 1.

A microscope image of the microprocessor is shown in Fig. 3a. The device is composed of 115 MoS_2_ transistors and measures—without bonding pads—0.6 mm^2^ in size. Circuit schematics for a D-Latch and the ALU are shown in Fig. 3b,c, respectively. The complete schematic is presented in Supplementary Fig. 1. A D-Latch is a bi-stable circuit that can be used as 1-bit data storage element, triggered by a CLK signal. It forms the basic building block of all our data registers (IR, AC and OR) and the PC. The ALU is a combinational logic circuit, entirely based on NANDs, that performs bitwise logic operations on 1-bit data. The additional input A/O signals the ALU which operation to perform. Measurements of the ALU output for different input logic states are presented in Supplementary Fig. 8.

**Figure 4:**
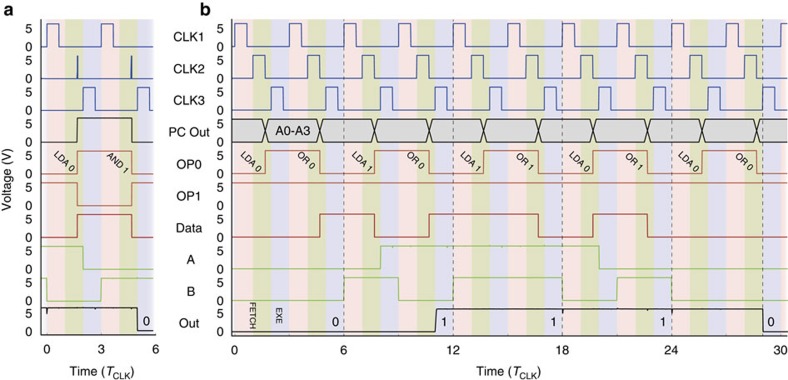
Device operation. (**a**) Waveforms measured on the chip when running the sample program, explained above. The CLK1, CLK2 and CKL3 signals were generated externally. Each instruction requires three CLK cycles, where 1/*T*_CLK_ is the CLK frequency. CLK2 pulses were made sufficiently short to trigger the level-sensitive input of the PC; this could be avoided by using a more complex master-slave design (Supplementary Fig. 9). A0 is the address supplied by the PC. OP0, OP1 and D0 denote the signals from the memory, where the former two are the instruction and the latter is the data. A and B are the input signals to the ALU, and OUT is the output of the device. *T*_CLK_=500 ms. (**b**) Results for a series of other calculations. In order to run the longer program, a 4-bit PC was implemented externally. The meaning of the curves is the same as in **a**, apart from A0 which schematically depicts here the 4-bit address signal (A0*–*A3). Again, the device is able to deliver the expected logic values, shown as numbers at the bottom.

We first verified the functionality of the microprocessor by running the example program from above and measuring waveforms at different locations on the chip (see Methods for measurement details). As shown in Fig. 4a, the device is indeed able to deliver the correct result, with excellent signal integrity and with rail-to-rail performance, proving the ability to cascade logic stages based on 2D semiconductors. To further demonstrate the operability of the device, we present in Fig. 4b the results from a series of logical disjunction operations. The match of measured and expected outputs shows again correct operation. As shown in Supplementary Fig. 10, the device proved to be functional at CLK frequencies of 50 Hz. This is by no means a limitation of the TMD material itself, but is caused by the limitations of our measurement setup. Ultimately, the speed is limited by the current-driving capability of the pull-up transistor, which is operated in the sub-threshold regime (*V*_GS_=0<*V*_T_) and acts as current source with *I*_D_≈0.55 μA. For a typical (external) capacitive load of *C*_L_≈1–10 pF, we estimate a maximum operation frequency of 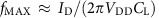
≈2–20 kHz (Supplementary Fig. 11). To increase *f*_MAX_, *I*_D_ could be increased by employing depletion-mode load FETs^20^, controlled chemical doping, improving the carrier mobility of the 2D semiconductor or just by reducing the transistor channel lengths.

## Discussion

In summary, we have reported a first step towards the development of microprocessors based on 2D semiconductors. The major challenge that we faced during device fabrication is yield. Although the yield for subunits was high (for example, ∼80% of ALUs were fully functional), the sheer complexity of the full system, together with the non-fault tolerant design, resulted in an overall yield of only a few per cent of fully functional devices. Imperfections of the MoS_2_ film, mainly caused by the transfer from the growth to the target substrate, were identified as main source for device failure. However, as no metal catalyst is required for the synthesis of TMD films^27^,^28^,^29^,^30^, direct growth on the target substrate is a promising route to improve yield. Besides that, we do not see any roadblocks that could prevent the scaling of our 1-bit design to multi-bit data. Our work demonstrates that integrated circuits consisting of 2D materials are a promising emerging technology.

## Methods

### Device fabrication

Fabrication (Supplementary Fig. 7) started with patterning of the bottom metal (gate) layer by electron beam lithography (EBL) and evaporation of Ti/Au (5/25 nm). A 22-nm-thick Al_2_O_3_ gate oxide was then deposited using atomic layer deposition, followed by a second lithography step and wet chemical etching in potassium hydroxide to define the via-holes that connect the bottom and top metal layers where necessary. Following the procedure described in ref. 29, a large-area MoS_2_ film was grown by CVD on sapphire and then transferred onto the target wafer. The film is continuous over an area of ∼50 mm^2^ with bilayer thickness and small multi-layer MoS_2_ islands and contaminations. The MoS_2_ film was characterized by atomic force microscopy and Raman spectroscopy (Supplementary Fig. 2). In a third EBL step, rectangular MoS_2_ channels were patterned and subsequently etched using Ar/SF6 plasma. Before lift-off, mild treatment of the sample in oxygen plasma was performed to remove the crust from the surface of the polymer mask. The top metal (drain/source contact) layer was then formed by another EBL process and subsequent Ti/Au (5/35 nm) deposition. The sample was finally annealed in vacuum at 400 K for several hours to remove adsorbants from the surface and reduce device hysteresis.

### Electrical testing

For testing, we generated the CLK signals externally, using a digital I/O card (National Instruments PCI-6229) in a computer. The same card was used for emulating the external memory. The device was supplied with *V*_DD_=5 V, and waveforms were recorded with a Semiconductor Parameter Analyzer (Agilent 4155C), connected to the probe tips of a wafer probe station (Lakeshore TTPX).

### Data availability

The data that support the findings of this study are available from the corresponding author on request.

## Additional information

**How to cite this article:** Wachter, S. *et al*. A microprocessor based on a two-dimensional semiconductor. *Nat. Commun.* **8,** 14948 doi: 10.1038/ncomms14948 (2017).

**Publisher's note**: Springer Nature remains neutral with regard to jurisdictional claims in published maps and institutional affiliations.

## Supplementary Material

Supplementary Information Supplementary Figures
