# Supplementary material for: A microprocessor based on a two-dimensional semiconductor
Source: Nat Commun. 2017 Apr 11;8:14948. doi: 10.1038/ncomms14948 (PMC5394242; doi:10.1038/ncomms14948)
Supplement: Supplementary Information — Supplementary Figures [file ncomms14948-s1.pdf]

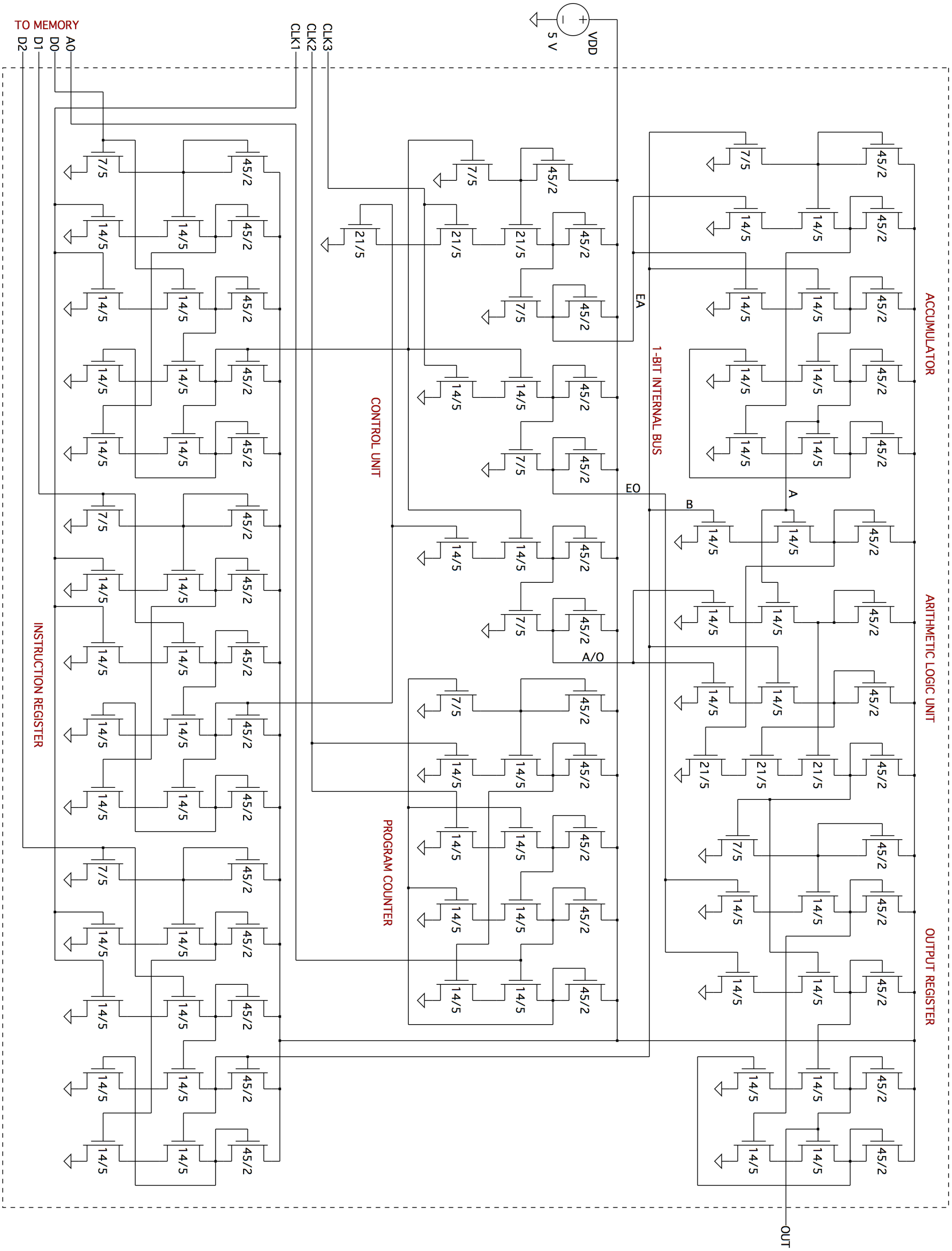

Supplementary Figure 1| Circuit schematic of the microprocessor.

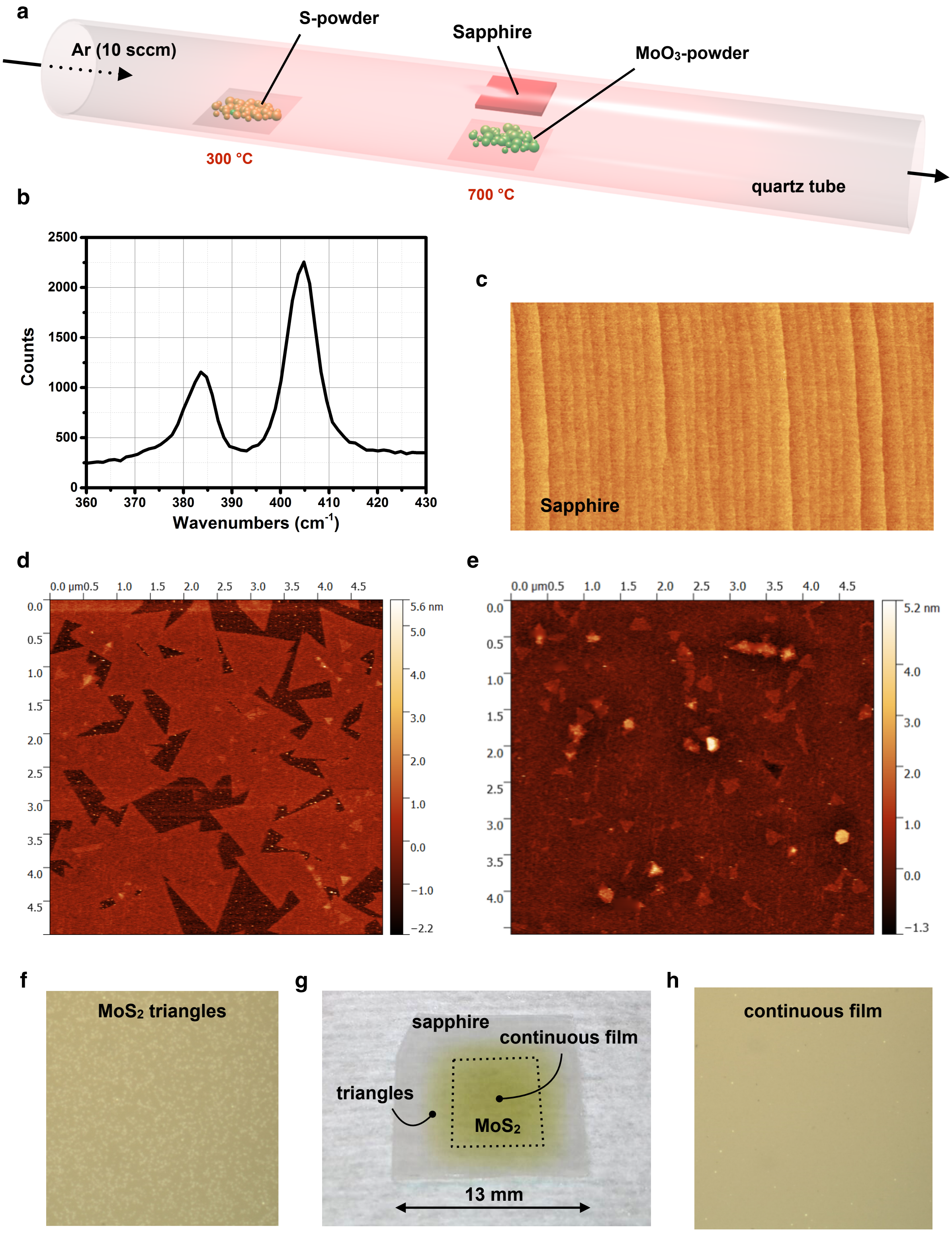

Supplementary Figure 2| MoS_2 film growth. (a) Schematic illustration of the growth setup. (b) Raman measurements, taken in the center of the sample, reveal that the MoS_2 film is a bilayer ($E_{2g} - A_{1g}$ spacing: 21.2 cm^{-1}). (c) AFM image of sapphire growth substrate. (d) The AFM image taken close to the edges of the sample shows triangular MoS_2 monolayer growth. (e) Towards the center of the sample, the triangles coalesce and form a continuous, polycrystalline film. (f) Microscope image of MoS_2 triangles. (g) Photograph of the MoS_2 layer grown on a sapphire. (h) Microscope image of continuous MoS_2 film.

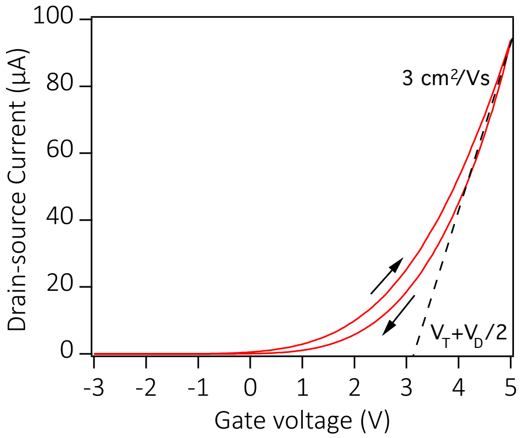

Supplementary Figure 3| Transistor transfer characteristic. Field-effect mobility ($\mu \approx 3$ $cm^2 V^{-1} s^{-1}$), hysteresis, and threshold voltage ($V_T \approx 0.65$ V) of MoS_2 FET. $V_D = 5$ V.

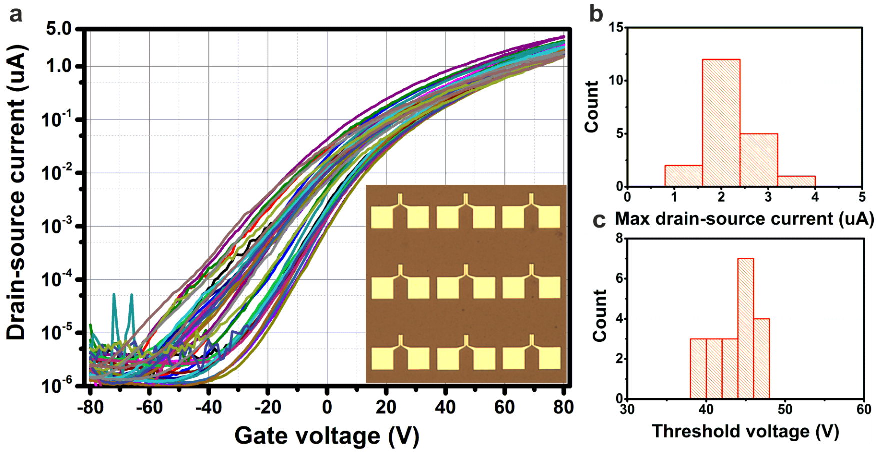

Supplementary Figure 4| MoS_2 film uniformity. To study the uniformity of our CVD-grown MoS_2 films, we fabricated an array of back-gated transistors over a ~3×3 mm^2 area on a Si/SiO_2 (300 nm) test wafer. (a) Transfer characteristics (scanned forth and back) of 20 devices (inset: microscope image, showing 9 devices). Histograms of (b) maximum drain-source current and (c) threshold voltage show good uniformity.

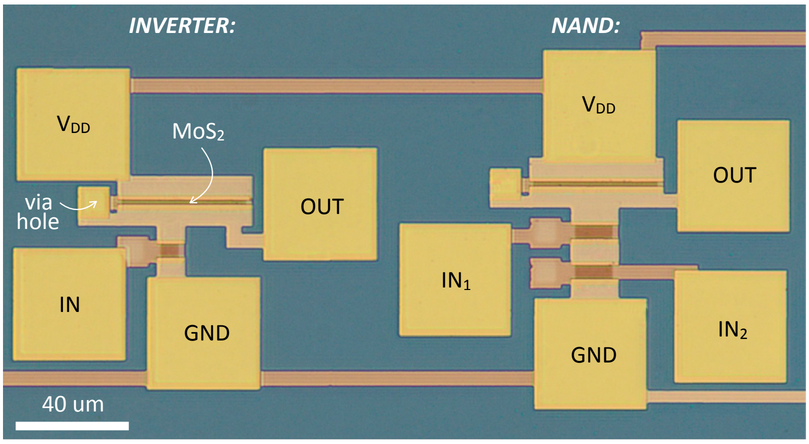

Supplementary Figure 5| Inverter and NAND logic stages. Micrographs of inverter (left) and NAND (right) test structures.

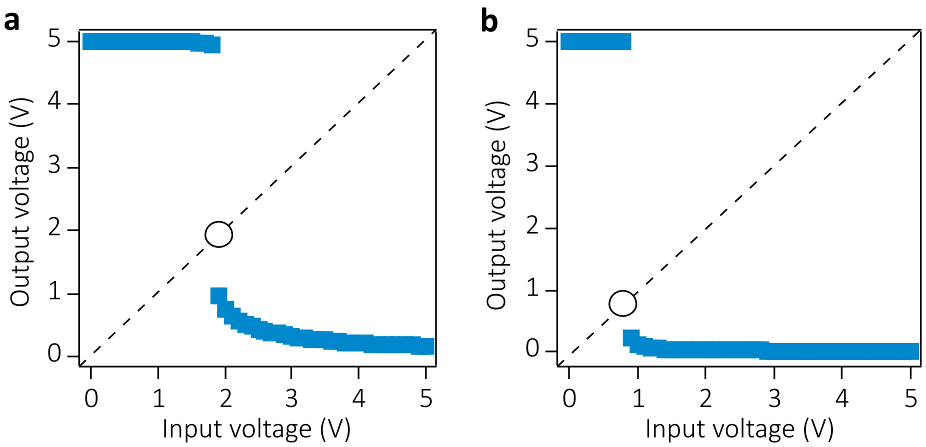

Supplementary Figure 6| Calculated inverter input-output characteristic. (a) Results for the asymmetric transistor design presented in the manuscript (Figure 2a). (b) If both transistors are implemented with same W/L-ratio, the switching threshold drops below 1 V. The circles show the switching threshold voltages V_{M}.

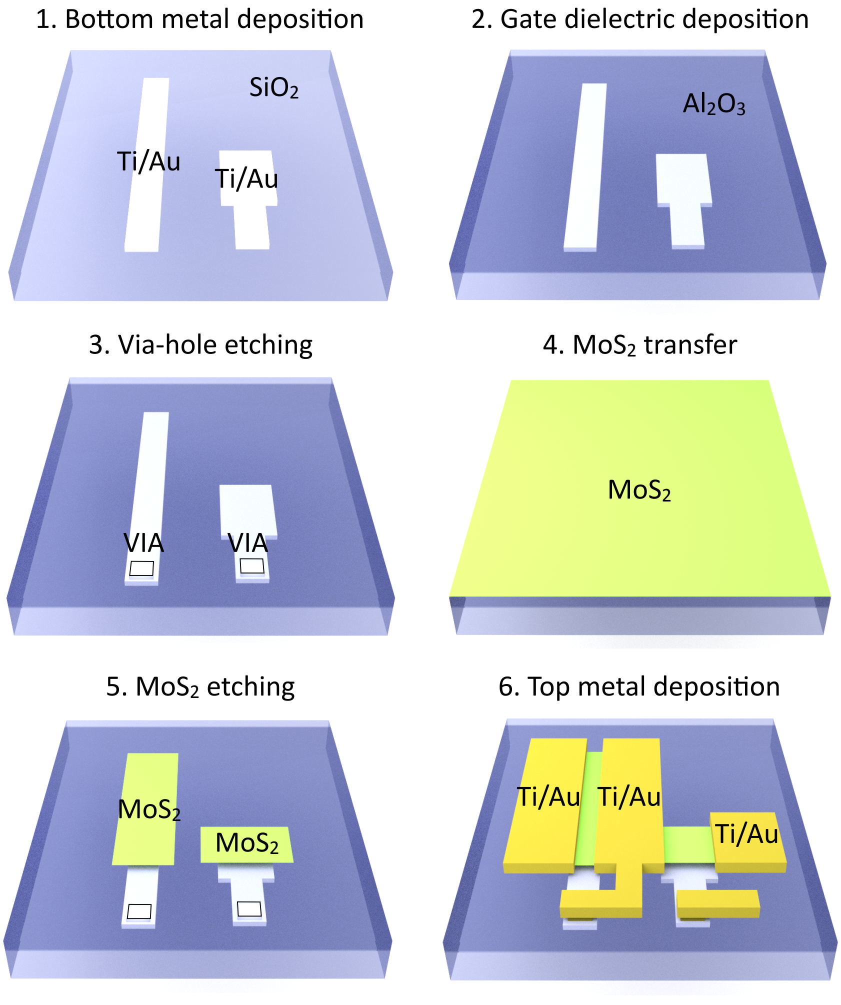

Supplementary Figure 7| Device fabrication process flow.

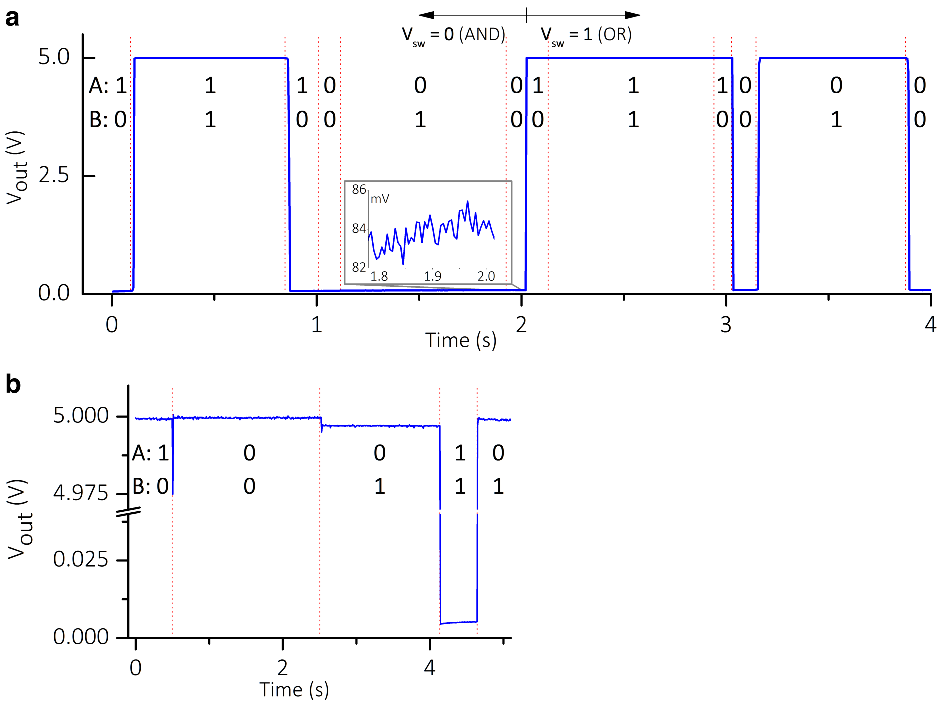

Supplementary Figure 8| ALU and NAND output voltage levels. (a) Time-domain measurements of ALU output voltage V_{OUT} for different A, B, and A/O input logic states (0, $V_{IN} =$ 0 V; 1, $V_{IN} =$ 5 V). (b) Same measurement for a NAND.

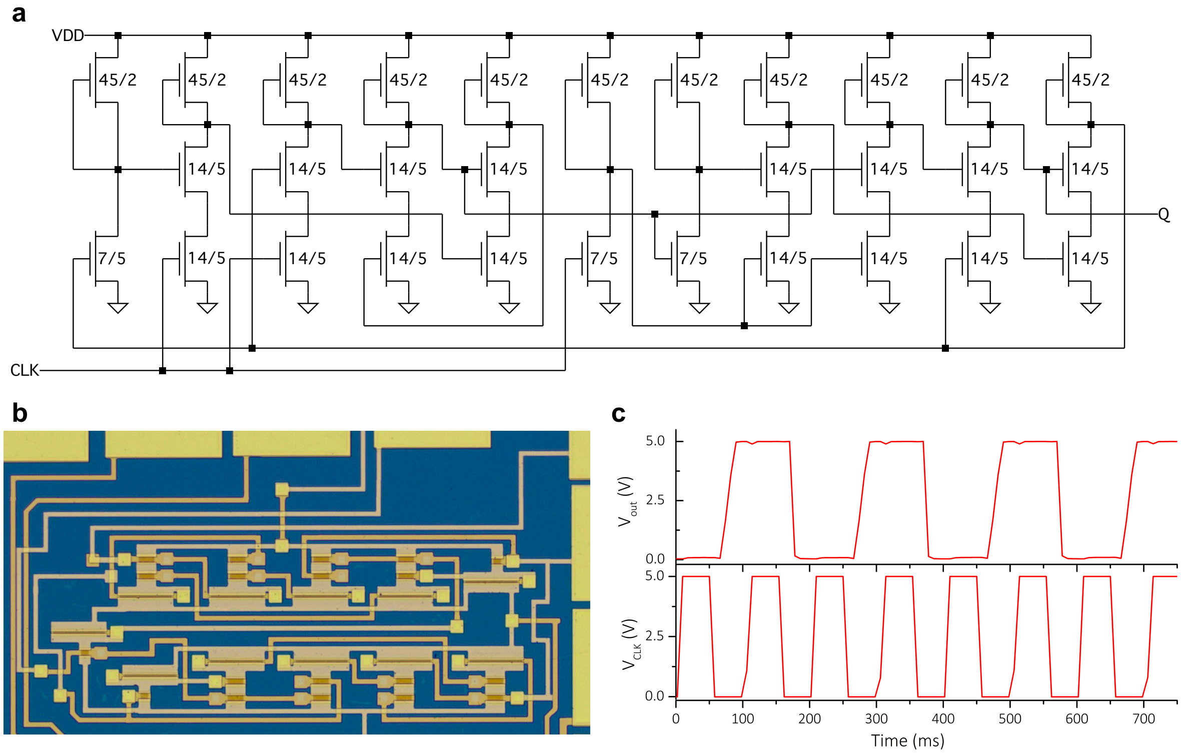

Supplementary Figure 9| Improved program counter. Program counter based on a master/slave flip-flop. (a) Circuit schematic, (b) microscope image, (c) device operation at $1/T_{CLK}$ = 10 Hz (bottom curve: input CLK signal, upper curve: output signal).

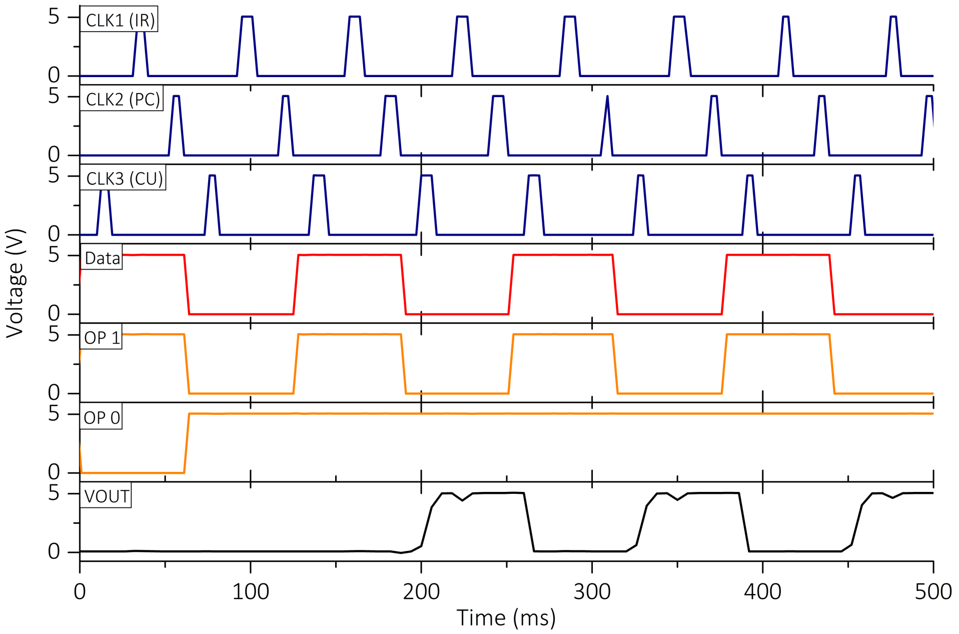

Supplementary Figure 10| Microprocessor waveforms. Operation of the microprocessor at $1/T_{\mathrm{CLK}} = 50$ Hz (limit of our measurement setup).

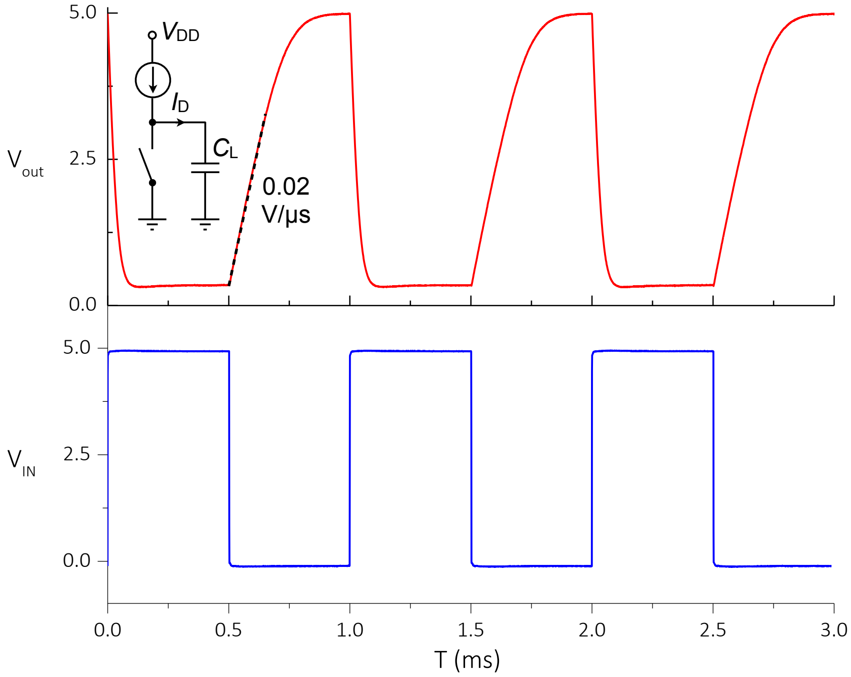

Supplementary Figure 11| Temporal characterization of inverter. In order to determine the ultimate speed limit of our NMOS logic stages, we investigated the temporal response of an inverter with external capacitive load of $C_{\mathrm{L}} = 30$ pF. It is limited by the current-driving capability of the load transistor, which is operated in the sub-threshold regime with $I_{\mathrm{D}} \approx 0.55$ μA (see inset). From the measurement we determine $\Delta V_{\mathrm{out}}/\Delta t \approx 0.02$ V μs^{-1}, in good agreement with the theoretically expected value of $\Delta V_{\mathrm{out}}/\Delta t = I_{\mathrm{D}}/C_{\mathrm{L}} \approx 0.018$ V μs^{-1}. If the capacitive load is reduced to $C_{\mathrm{L}} \approx 1$–10 pF, we can thus expect a maximum operation frequency of $f_{\mathrm{MAX}} \approx 2$–$20$ kHz.

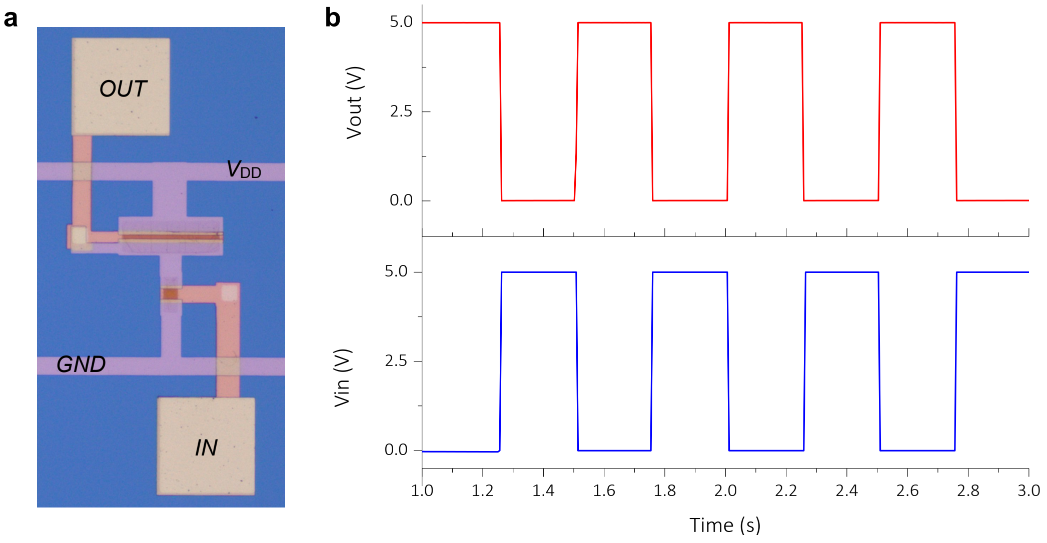

Supplementary Figure 12| Optical lithography. Although our microprocessor was fabricated with electron beam lithography, we also developed a technology for the fabrication of logic stages with standard optical lithography. A microscope image of a so obtained inverter and corresponding waveforms are shown in (a) and (b) respectively. The process flow is similar to that described in the Methods section, apart from via-hole etching which was done by MIF developer instead of KOH.
